# Tailoring cells for clinical needs: Meeting report from the Advanced Therapy in Healthcare symposium (October 28–29 2017, Doha, Qatar)

**DOI:** 10.1186/s12967-018-1652-y

**Published:** 2018-10-10

**Authors:** Sara Deola, Bella S. Guerrouahen, Heba Sidahmed, Anjud Al-Mohannadi, Muhammad Elnaggar, Ramaz Elsadig, Essam M. Abdelalim, Goran Petrovski, Massimo Gadina, Adrian Thrasher, Winfried S. Wels, Stephen P. Hunger, Ena Wang, Francesco M. Marincola, ATH Consortium, Maria Ester Bernardo, Patrizia Comoli, Kevin Curran, Graham Davies, Gianpietro Dotti, Mamoun Elawad, Soldano Ferrone, Antonia Follenzi, Ramsay Fuleihan, Amar Gajjar, Amel Hassan, Ziyad Hijazi, Katarina Le Blanc, Michele Maio, Eoin McGrath, Peter Parham, Lorenzo Piemonti, Matthew Porteus, Waseem Qasim, Raya Saab, David Stroncek, Fewzi Teskrat, Abdalla E. Zarroug, Cristina Maccalli, Chiara Cugno

**Affiliations:** 1 Research Department, Clinical Research Center, Sidra Medicine, Doha, Qatar; 2 Diabetes Research Center, Qatar Biomedical Research Institute, Hamad Bin Khalifa University, Qatar Foundation, Education City, Doha, Qatar; 3 Endocrinology Unit, Sidra Medicine, Doha, Qatar; 4 National Institutes of Health, Bethesda, MD USA; 5 UCL Great Ormond Street Institute of Child Health, London, UK; 6 Georg Speyer Haus, Institute for Tumor Biology and Experimental Therapy, Frankfurt, Germany; 7 Children’s Hospital of Philadelphia, Philadelphia, USA; 8 Immune Oncology Discovery and System Biology, AbbVie, Redwood City, CA USA; 9 Refuge Biotechnologies Inc., Menlo Park, CA USA

**Keywords:** Personalized medicine, Cell therapy, Gene therapy, Stem cells, Autoimmunity, Oncology, Hematology, Preclinical, Clinical trials

## Abstract

New technologies and therapies designed to facilitate development of personalized treatments are rapidly emerging in the field of biomedicine. Strikingly, the goal of personalized medicine refined the concept of therapy by developing cell-based therapies, the so-called “living drugs”. Breakthrough advancements were achieved in this regard in the fields of gene therapy, cell therapy, tissue-engineered products and advanced therapeutic techniques. The Advanced Therapies in Healthcare symposium, organized by the Clinical Research Center Department of Sidra Medicine, in Doha, Qatar (October 2017), brought together world-renowned experts from the fields of oncology, hematology, immunology, inflammation, autoimmune disorders, and stem cells to offer a comprehensive picture of the status of worldwide advanced therapies in both pre-clinical and clinical development, providing insights to the research phase, clinical data and regulatory aspects of these therapies. Highlights of the meeting are provided in this meeting report.

## Introduction

The Advanced Therapies in Healthcare (ATH) symposium (http://events.sidra.org/event/advanced-therapies-in-healthcare/), held in Doha, Qatar (October 2017), offered an outstanding combination of talks, capturing a broad range of topics in the area of advanced therapies worldwide (Table 1).

**Table 1:** Summary of speakers and topics

Speaker	Title	Clinical Application
Adrian Thrasher	Evolving gene therapy for primary immunodeficiency	Primary immunodeficiencies
Matthew Porteus	Genome editing of stem cells to cure genetic diseases of the blood and immune system	Sickle cell disease SCID-X1
Waseem Qasim	Gene engineered immune therapy	Cancer (B-ALL)
Ramsay Fulham	Stem cell transplantation (reduced intensity conditioning) and obstacles and new approaches to Gene Therapy for hyper IgM syndrome	Hyper IgM syndrome
Antonia Follenzi	Cell and gene therapy for hemophilia A	Hemophilia A
Katarina Le Blanc	MSC in clinical trials for type 1 diabetes	Diabetes
Maria Ester Bernardo	Autologous bone marrow-derived mesenchymal stromal cells in the treatment of fistulising Crohn’s disease	Crohn disease
Patrizia Comoli	Antigen-specific T cell therapy in hematology/oncology	Viral infections in immunosuppressed patients (EBV, CMV, HHV6, BK, JCV)
Essam Abdelalim	Pluripotent stem cell-derived pancreatic beta cells: therapeutic potential and challenges in diabetes treatment	Diabetes
Lorenzo Piemonti	Toward beta cell replacement for diabetes	Diabetes
Peter Parham	HLA and KIR in human health and survival	NK mediated immune responses
Graham Davies	Thymus transplantation	Primary thymic disorders
Mamoun Elawad	Allogeneic HSCT for inflammatory gut diseases	Inflammatory gut diseases bowel disease
Amel Hassan	Hematopoietic stem cell transplant for PID	Primary immunodeficiencies
Raya Saab	Genomics of childhood cancer	Pediatric cancers
Amar Gajjar	Molecularly directed therapy for pediatric brain tumors	Pediatric cancers
Massimo Gadina	Inhibition of cytokine signaling in autoimmune and inflammatory diseases: the coming of age of JAK inhibitors	Autoimmune diseases
Holm Uhlig	New non-transplant approach in treating CGD and primary neutropenia	Inflammatory bowel disease, CGD, primary neutropenia
Soldano Ferrone	HLA antigens and immunotherapy of malignant diseases	Cancer (melanoma, head and neck squamous cell carcinoma, breast cancer)
Michele Maio	Immune checkpoint inhibitors	Cancer (melanoma, lung carcinoma, colorectal cancer, mesothelioma)
Francesco Marincola	Addressing cancer responsiveness immunotherapy	Cancer (immune responsiveness)
Giampietro Dotti	Car-T cells: from bench to bedside	Cancer (CD19+ cancers, glioblastoma, ductal adenocarcinoma, ovarian cancer, neuroblastoma)
Kevin Curran	CAR T-cell for cancer immunotherapy	Cancer (B-ALL)
Stephen Hunger	Treatment of relapsed pediatric acute lymphoblastic leukemia. The promise of CAR T-cell therapy	Cancer (B-ALL)
David Stroncek	CAR-T cells: promise and problems	CAR-T cell manufacturing
Winfried Wels	CAR-engineered NK cells for adoptive cancer immunotherapy	Cancer (Glioblastoma)
Ziyad Hijazi	Current state of percutaneous pulmonary valve replacement	Cardiac surgery
Goran Petrovski	Closing the loop in diabetes: the impact of sensor augmented pump	Diabetes
Abdalla Zarroug	Intrauterine surgery	Fetal surgery
Fawzi Teskrat	Inspection of ATMP’s activities	Quality and compliance
Eoin McGrath	JACIE accreditation: an overview	Quality and compliance
Eoin McGrath	New therapies: adapting standards and regulations to immune effector cells	Quality and compliance

This meeting report summarizes the key advancements presented in the symposium, in the areas of gene therapy, cancer immunotherapy, cell therapy/adoptive cell therapy, diabetes and general therapeutic techniques.

## Gene therapy

During last decade, the field of gene therapy has enormously progressed, regaining its “fame” after the whole world held its breath for the first viral-insertion oncogenesis events. Cases of overt leukemias in X-linked Severe Combined Immune Deficiency (SCID-X) immunodeficient patients treated with retroviral vectors-corrected stem cells placed the whole field in the eye of the storm in the early 2000s [1].

In 2016, after proving its safety with years of studies on clonal insertion, of vector improvements and robust follow-ups, gene therapy matured, from the infancy of a few case reports cured by gifted scientists, into the production of its first commercial drug.

Gene therapy also changed the whole concept of a “drug”, introducing the frame of a “process”, entailing a few days of high-level cell manufacturing, and resulting in a therapeutic product that can be defined as a “living drug” [2].

The first gene therapy drug to hit the market was Strimvelis, a gamma-retroviral based hematopoietic stem cell gene therapy process for ADA-SCID patients [3, 4].

The concomitant breakthrough of CAR-T cells launched two more commercial drugs in the following years. CAR-T cells targeting the CD19 molecule are now available in the market for relapsed/refractory B-ALL and diffuse large B cell lymphoma treatment in two different flavors: Kymriah (Tisagenlecleucel, gamma-retroviral based) and Yescarta (axicabtagene ciloleucel, also called axi-cel, lentiviral based).

### A brief historical overview: gene therapy of primary immune deficiencies

Fifty years ago, SCIDs were fatal diseases, and only after the introduction of allogeneic hematopoietic stem cell transplantation (HSCT) and exogenous enzyme replacement the first clinical responses were gained. Nowadays a stable cure has been achieved through gene therapy approaches.

Long-term disease-free survival of different Adenosine Deaminase (ADA)-SCID gene therapy clinical trials in Europe and US ranges from 50 to 83% [5].

The oncogenic adverse events occurring in SCID-X [1] paved the way to the development of refined vector technologies, including the use of self-inactivating gamma-retroviral and lentiviral vectors in which the powerful viral enhancer sequences are deleted. Clinical trials with these vectors have now been reported, including SCID-X (RV; NCT01410019, NCT01175239, NCT01129544), Wiskott-Aldrich syndrome (WAS; LV; NCT01347242), ADA-SCID (LV; NCT01380990 and NCT02234934) and Chronic Granulomatous Disease (LV; NCT02234934, NCT01347346), with encouraging preliminary results of enhanced safety and long-term efficacy [6]. Some of these products are being lined up for regulatory licensing.

Furthermore, new technologies, including homologous recombination or gene repair to accurately correct genetic mutations or insert therapeutic sequences, are on their way towards clinical translation.

### Gene therapy of hemophilia A

Hemophilia A is a monogenic X-linked disease caused by the lack of Factor VIII, and it is very life-compromising in its severe form. A very modest protein product of the missing gene would be able to functionally correct the disorder, making the disease an optimal target for gene therapy.

Adenoviral vector (AAV)—mediated gene therapy targeting the liver was able for the first time in clinical trials to achieve a long-term clinical benefit both for Hemophilia A and B—with some “caveats”: candidate patients should not possess natural anti-AAV antibodies and—the liver being very immunogenic—signs of transaminitis. These clinical conditions will lead to an immune rejection of the transgene expressing cells and, thus, will immediately require high dose steroids to preserve the integrity of the therapy.

Lentiviral vectors may be able to deliver FVIII transgene to target cells, with less immunogenicity. Liver endothelial sinusoidal cells and Kupffer cells are the main FVIII producers in the liver. By using cell-specific promoters, optimized with miRNA, a tolerizing pattern towards the transgene was shown in mice, mediated by T-regs intervention. Moreover, by using an endothelial specific promoter (Vascular Endothelial Cadherin 5), long term transgene expression in mice is observed with no immune responses [7]. Novel gene transfer technologies include a striking gene delivery “immune-protected” system where gene modified cells would release the transgene through a small implantable device (Cell Pouch™ http://www.sernova.com/technology/).

### Gene editing of stem cells (sickle cell disease, SCID-X1)

Gene editing represents a promising approach to improve the clinical efficacy of gene therapy. The first CRISPR/Cas9 gene-editing platform for repairing the β-globin gene in hematopoietic stem cells was reported in 2016 [8]. A combination of Cas9 ribonucleoproteins and AAV donor DNA delivery was able to precisely repair the sickle cell disease causing E6V mutation in the β-globin gene in patients’ hematopoietic stem cells. The gene expression recovery was highly efficient, with reproducibility and acceptable low off-target reactivity among patients.

Further optimization of the delivery method by using a High Fidelity Caspase 9 technique (from Integrated DNA Technologies, IDT) significantly reduced the off-target editing, while maintaining the on-target performance, with a final rate of 40–50% gene-corrected cells [9].

Similar striking results were also obtained in SCID-X1 immunodeficiency through repairing the IL-2 gamma-chain receptor in stem cells. Two Phase I/II trials are to commence in 2019 in Stanford for both hemoglobinopathies and primary immunodeficiencies.

### Gene therapy of cancer—the CARs (T and NK)

#### Clinical efficacy of CARs

Survival rates for children and adolescents with acute lymphoblastic leukemia (ALL) have improved steadily over the past 5 decades, with 5-year overall survival (OS) now exceeding 90% [10–12]. However, high risk subgroups of newly diagnosed ALL patients can be identified that have an OS < 60% [13] due to high rates of disease relapse, particularly bone marrow relapse [14]. Furthermore, only a small portion of children and adolescents that experience a 2nd relapse, or relapse after a prior allogeneic HSCT, are cured by salvage therapies. Intensification of standard cytotoxic chemotherapy agents and/or HSCT have not significantly improved OS for these very high risk subgroups of newly diagnosed and relapsed ALL, identifying a major unmet medical need.

The Children’s Hospital of Philadelphia (CHOP)/Univeristy of Pennsylvania (Penn) group is among the first pioneers of adoptive cell therapy treatment of ALL patients with CTL019 (tisagenlecleucel) CAR-T cells. Among an initial cohort of 25 children recruited at CHOP and 5 adults recruited at the University of Pennsylvania Hospital (NCT01626495 and NCT01029366) with relapsed/refractory B-ALL, 90% attained complete responses (CR) [15]. All patients developed the Cytokine-Release Syndrome (CRS), which was of a high grade (3–4) in 27% of cases. Patients with severe CRS were rescued successfully with the interleukin-6 receptor monoclonal antibody (mAb) tocilizumab. With a follow up period of 2–24 months, half of the cohort remained in complete remission, with most receiving no further leukemia therapy.

The pediatric cohort of the clinical trial was increased to 60 subjects and was followed over time. The CR rate was 93%. After a median follow up of 15 months (1–48 mos), 24 patients out of 60 showed continuous remission over 1 year in duration; moreover, 19 of these patients did not require other therapies.

These results are particularly remarkable and unprecedented as 2/3 of treated patients had undergone a prior allogeneic HSCT, meaning that CAR-T cell strategy can restore sustained anti-cancer immune responses even after failure of graft versus leukemia (GVL) responses.

A multicenter Phase II study (ELIANA; NCT02435849) with CTL019 CAR-T cells has been performed enrolling patients in US Canada, Europe, Australia and Japan [16]. Among 75 patients with sufficient follow-up, over 80% of patients achieved remission, which was complete in three-quarters of patients and was with incomplete blood count recovery in the remaining quarter. Patients with complete clinical responses had no detectable minimal residual disease. Disease-free survival was 73% at 6 months and 50% at 12 months, and OS was 90% at 6 months, and 76% at 12 months. This clinical study led to the first approval by the Food and Drug Administration (FDA) of tisagenlecleucel.

Another Phase I Multicenter Trial administered CD19 CAR-T cells to young adult patients with relapsed/refractory B-ALL (NCT01860937). CAR-T cells were manufactured at the Memorial Sloan Kettering Institute, NY, USA, and patients were recruited and treated at the Dana Farber Cancer Institute and Boston Children’s Hospital, Boston, USA. In 24 evaluable patients, CR rate was 75%, significantly better after high dose cyclophosphamide (Cy) vs low dose chemotherapy, while the addition of Fludarabine to the therapeutic regimen did not provide significant benefits. Pre-treatment molecular remission was decisive for OS, granting a plateau of survival over 500 days for about 75% of this patient group.

Natural killer (NK) cells represent a valuable innate immune cell population for adoptive cell therapy, but clinical experience with CAR-engineered NK cells is still limited. In cancer patients, NK cells, like other immune cells, are often functionally compromised due to the immunosuppressive features of the tumor microenvironment (TME). Hence, for adoptive cell therapy, donor-derived allogeneic NK cells are preferable since they do not recognize tumor cells as “self”, thereby bypassing inhibitory signals. It is possible to exploit the therapeutic efficacy of NK cells through expression of CARs, using the NK-92 cell line. This clinically applicable cytotoxic cell line provides an unlimited source of effector cells and holds potential for development as a standardized “off-the-shelf” therapy [17].

GMP-compliant protocols for vector production, lentiviral transduction and expansion of a CAR-engineered cell clone (NK-92/5.28.z) were established which carries a HER2 (ErbB2)-specific CAR that harbors a composite CD28-CD3ζ signaling domain [18]. Functional analysis of NK-92/5.28.z cells revealed high and stable CAR expression, and selective cytotoxicity against HER2-positive tumor cells of different origins. Importantly, upon activation, NK-92/5.28.z cells could secrete high levels of proinflammatory factors without upregulating immune checkpoint molecules. CAR-mediated cytotoxicity was not affected by immunosuppressive factors such as a hypoxic environment and TGF-β. With the aim of developing NK-92/5.28.z cells for the treatment of HER2-positive glioblastoma multiforme (GBM), the reactivity of these cells against GBM cell lines and primary GBM cultures was evaluated, showing selective HER2-dependent cell killing in vitro. Potent in vivo anti-tumor activity of NK-92/5.28.z was observed in orthotopic GBM xenograft models in NOD scid common gamma chain knockout (NSG) mice, leading to a marked extension of OS upon repeated stereotactic injection of CAR-NK cells into the tumor [17, 19]. In immunocompetent transplanted syngeneic GBM animals, local therapy with NK-92/5.28.z cells resulted in induction of endogenous antitumor immunity and long-term protection against tumor rechallenge at distant sites. These results suggest that adoptive cell transfer of HER2-specific NK-92/5.28.z cells represents a promising immunotherapy approach for GBM. A Phase I clinical trial investigating intracranial injection of NK-92/5.28.z cells as a treatment for recurrent HER2-positive GBM is ongoing (NCT03383978).

#### Side effects of CARs

CARs have also of course their limits and pitfalls. The first side effect is represented by CRS, caused by a large, rapid release of cytokines into the blood from immune cells affected by the immunotherapy: all patients in the initial CHOP/Penn cohort and 80% of patients of Memorial Sloan Kettering Institute developed some degree of CRS, with a wide range of severity. At least 50% of patients required admission to Intensive Care Units, and 15–30% of them battled against life threatening complications. The toxicities of CAR T-cell therapy are not only acute, but also include B-cell lymphopenia, since normal B-cells express CD19. The lymphopenia is associated with low levels of immunoglobulin production such that patients usually required regular intravenous immunoglobulin infusions to restore a protective humoral immunity, while CAR-T cells are detectable in their body.

To increase the safety of CAR-T cells, a “safety switch” can be used [20, 21], allowing the termination of CAR-T cell survival in case of toxicity.

The potent iC9 system is based on a modified human caspase-9 fused to the human FK506 binding protein (FKBP), sensitive to AP1903/Rimiducid drug. After 1 dose drug delivery, > 85–90% of iC9-transduced T cells are rapidly eliminated, with the advantage of leaving behind a residual efficient population that can be re-expanded in the event of a tumor relapse [22].

After the construct was validated in a mouse model, the LC Cancer Center University of North Carolina—Chapel Hill initiated a clinical trial to evaluate the safety and efficacy of this therapy in N = 40 patients with relapsed/refractory B-ALL (NCT03016377).

#### Target “antigens” for CARs

Almost 20 clinical trials ongoing worldwide are trying to expand CARs cell therapy in solid cancer diseases, including breast cancer, nasopharyngeal carcinoma, lung cancer, cervical cancer, colorectal cancer, pancreatic cancer, head and neck cancer, and brain cancers (https://clinicaltrials.gov/ct2/results?cond=Cancer&term=CAR-T&cntry=&state=&city=&dist=&Search=Search).

Chondroitin sulfate proteoglycan 4 (CSPG4) is a protein overexpressed by tumors with different histological origins, such as GBM, astrocytoma, head and neck cancers, renal clear cell carcinoma, triple negative breast cancer and melanoma. Particularly in GBM, this antigen has been detected in cancer stem cells and tumor-associated vessels. The intracranial injection of CSPG4.CAR-T cells successfully controlled tumor growth in mice orthotopically xenografted with GBM cells. A “first in man” Phase I clinical study is being planned in LC Cancer Center University of North Carolina—Chapel Hill for GBM patients, treated with CSGP4.CAR T cells, with the iC9 safety switch.

Another appealing target molecule for CAR-T cells is represented by B7-H3 which is a member of the B7 family aberrantly expressed by cancer and stroma fibroblasts in ovarian and pancreatic cancers, GBM and several other type of tumors. Promising results have been obtained in mouse models using anti-B7-H3 CAR-T cells. A Phase I clinical study for patients with ovarian cancer is also planned for this target molecule.

Moreover, neuroblastoma patients are benefitting from T-cells engineered with a 3rd generation (CD28-OX40) anti-GD-2 CAR-T construct, associated with an iC9 suicide safety switch, in the GRAIN study (NCT01822652), sponsored by Baylor College of Medicine. Interim results show that three out of 11 patients (27%) achieved a CR, however only 1 patient showed durable responses. The study is also aimed at comparing the clinical efficacy of the combination regimen of CAR-T cells with one anti-PD-1 agent, Pembrolizumab, which usually is not clinically effective in patients with this type of tumor.

#### Target “patients”

In front of an urgent clinical need, as often is the case of overt relapses unresponsive to salvage therapies, and when patients are not adequate for leukapheresis self-donation, the only available starting material to generate CAR-T cells is represented by third party blood cells.

To overcome the immunological barrier linked to the CAR-T cell design, some scientists are developing a combined approach with a 2-step engineering method: the insertion of CAR-T construct, plus gene editing to eliminate the native T-Cell Receptor (TCR) of T-lymphocytes. This strategy will reduce the possibility of causing Graft versus Host Disease (GvHD) and will represent the generation of “off-the-shelf” CAR-T cells.

CAR-T cells engineered to lack CD52 and TCR antigens through TALEN-mediated gene editing- “Universal” CD19-targeting cells (U-CART19) are now available in a Phase I study at the UCL/Great Ormond Street Hospital, in collaboration with Belgium and France (NCT02808442). The CAR product was launched after the successful application of a pilot study at UCL. In two cases of ALL pediatric patients who relapsed after allo-HSCT, the U-CART19 therapy was able to regain ALL molecular remission after other salvage therapies—including Blinatumomab—failed. Patients successfully underwent a second allo-HSCT thereafter.

Gene edited U-CART showed encouraging results on remission rates while their long-term safety and efficacy are still under evaluation.

#### Relapse after CAR-T cell therapy

Mechanisms of immune evasion are common in tumor cells to impair anti-tumor immune responses [23]. One of these mechanisms is represented by immune selection of tumor cells negative for the expression of defined antigens [24]. CAR-T cells, through targeting one single antigen, are not very versatile and became ineffective in tumor recognition upon failure of tumor cells to express a specific antigen. The strategy to develop CAR-T cells targeting multiple tumor antigens seems the best one to overcome this issue [25].

Another factor limiting CAR-T cells’ anti-tumor activity is their persistency. CAR-Ts with long lasting “central memory” phenotype [26] showed the highest persistence in vivo, therefore it is crucial to strategize a protection from their late stage of cellular differentiation [27]. Long lasting persistency may be also hampered by immune-mediated targeting of murine antigens in the construct. The CTL119-CAR-T cell construct, which uses a humanized version of the B-cell moiety, has been tested at CHOP/Penn with a 100% CR rate in 22 Relapsed/Refractory CD19^+^ B-ALL and B-cell lymphomas. Most strikingly, 15 patients could be re-treated after a first failure of CAR-T cells and achieved another 60%, with a 1-year relapse free survival of 52%. Taken together, these promising results are in favor of the clinical efficacy of CAR-T cells. Given the remarkable activity of CAR T-cells for ALL patients, it will be critical to develop clinical trials in patients with earlier stages of disease, including very high risk ALL in first remission and high risk subsets of ALL at first relapse.

#### Manufacturing of CAR-T cells

Examples of clinical grade CAR-Ts production include: CD19 and CD22 for ALL and lymphomas (and combinations: CD19/22, CD19/20), anti-GD2 for osteosarcoma and neuroblastoma, anti-BCMA for multiple myeloma, anti-CD30 for Hodgkin and non-Hodgkin lymphoma, anti-CCR4 for T cell malignancies and many others [28].

Most CAR-T cells are currently produced using leukocytes collected from the patient who is to receive the therapy. The use of autologous cells to manufacture the CAR T cells present unique challenges related to cell production which include:

- The production of several lots of small quantities of cells may cause highly manufacturing costs;
- Differences among subjects’ in terms of biological variations, underlying disease and related therapeutic treatments lead to product variability;
- Collection and manufacturing failures impede the treatment of some of the candidate patients.

In order to optimize the yield of CAR-T cell recovery, the enrichment of T cells from starting material (leukapheresis) is desirable. In particular, the use of CD4–CD8 binding magnetic beads to select T lymphocytes grants a better product consistency, even if increasing the manufacturing costs and complexity. Following T cell enrichment, the transduction phase is particularly critical. Important factors include the choice of vector, quantity of vector with respect to cell number, and time and type of cells’ exposure to the vector. Finally, appropriate GMP compliant quality controls are needed.

## Cancer immunotherapy

Increasing interest in the field of immunotherapy has been displayed during the last decades thanks to novel breakthroughs, leading this strategy to become another important pillar of cancer therapy together with surgery, chemotherapy, radiotherapy and targeted therapy. Monoclonal antibodies agonistic of immune checkpoints revealed to offer durable clinical responses in cancer patients with advanced cancers that are usually resistant to standard therapy, such as melanoma and non-small-cell lung cancer.

### A brief historical overview

Immunotherapy is based on the idea that a patient’s immune system can be stimulated or enhanced to attack tumors. One of the first examples of the principles of immunotherapy take us back to 1891, when William B. Coley, a bone sarcoma surgeon induced an infection by injecting streptococcal organisms into a patient with inoperable cancer [29]. Despite some degree of toxicity, he observed a regression of the tumor. In 1900, Paul Ehrlich postulated that if a drug is linked to a toxic chemical, it would act like a missile to inactivate unknown invaders, including cancer. This scientific theory of “magic bullets” has later inspired the scientific community to explore numerous molecular cancer therapeutics. In 1975, the hybridoma technology enabled the production of mAbs specific for a single epitope. mAbs against a specific cancer cell protein have been humanized to improve safety and efficacy, and in 1997, Rituximab, was the first mAb to gain FDA approval for the indication of relapsed or refractory, CD20 positive, B-cell, low-grade or follicular non-Hodgkin’s lymphoma.

### Cancer immunoediting

Changes in the immunogenicity of tumors occur due to the anti-tumor response, and the outcome is the generation of immune-resistant variant; this is one of the mechanisms leading to “cancer immunoediting”. Immunoediting is a dynamic process, composed of three sequential phases: elimination, equilibrium, and escape [30]. During the elimination phase, the innate and adaptive immunities work together, aiming to destroy the developing tumor. At the end of this phase, the host remains either free of cancer, or may enter an equilibrium phase if a rare cancer cell variant is not destroyed. During this state, adaptive immunity actors (among others: T cells, IL-12, and IFN-γ) are required to maintain tumor cells in a state of functional dormancy. This phase may also represent an end stage of the cancer immunoediting process. But, due to a constant immune selection pressure, tumor cell variants may emerge that are no longer recognizable by adaptive immunity (antigen loss variants or defects in the machinery of antigen processing and presentation). They may also become insensitive to immune effector mechanisms or induce an immunosuppressive state within the tumor microenvironment, entering a phase of escape, in which their outgrowth is no longer blocked by immunity.

### HLA antigens and immunotherapy of malignant diseases

Immune checkpoint inhibitors target co-stimulatory (agonistic) or inhibitory (antagonistic) molecules expressed on the surface of immune cells rather than on the surface of cancer cells, but the malignant transformation is often associated with defects in surface presentation of tumor antigen-derived peptides by the HLA class I antigen-processing and presenting machinery (APM), which is critical for T-cell adaptive immune responses against tumors [31, 32]. In a normal state, HLA class I antigen processing involves the proteasome complex for degradation of the tumor antigen into amino acid peptides fragments. The generated immunogenic tumor antigen-derived peptides are translocated across the endoplasmic reticulum membrane via the ATP-dependent heterodimeric transporter associated with antigen processing (TAP) complex. The assembly of newly synthesized HLA class I heavy chain molecules with β2-microglobulin (β2-m) and tumor antigen-derived peptides is assisted by transient interactions with calnexin, calreticulin, ERp57 and tapasin [33]. The resulting trimers are transported from the ER to the plasma membrane via the trans Golgi and can be presented to cytotoxic T lymphocyte (CTL) or NK cells. Abnormalities of the APM machinery can take place at the genetic (molecular defects of the heavy chain, immunoproteasome subunit, transporter molecule and β2-m genes) or epigenetic level [34]. Epigenetic modulation in gene expression can underlie HLA class I APM component defects in malignant cells and includes gene silencing by methylation and modification of chromatin structure by histone deacetylation. In some cases, strategies like pharmacologic agents inducing DNA hypomethylation or inhibition of histone deacetylation can counteract those epigenetic mechanisms and restore immunogenic antigen presentation, thus significantly increasing the effectiveness of cytotoxic T lymphocyte (CTL) response [34]. HLA class I APM component expression and/or function can also be affected by the over-activation of multiple oncogenic downstream pathways, such as MAPK in several tumor systems, including melanoma [35], and epidermal growth factor receptor (EGFR) in head and neck cancers. The effectiveness of CTL response can be restored via EGFR blockade by cetuximab, which triggers the transcription factor STAT1 to mediate the synthesis of HLA class I molecules and APM components [36, 37].

### Immune checkpoint inhibitors

The generation and maintenance of immune responses are controlled by a number of coinhibitory as well as costimulatory signals that can modulate T-cell receptor-engagement and its dependent T-cell activation and proliferation (CTLA-4, PD-1, B7-1, LAG-3, CD40L, CD137, OX40, and CD28) [38].

Cytotoxic T-lymphocyte antigen 4 (CTLA-4)—a CD28 homologue—is expressed mainly on the surface of activated T-cells and T-regulatory cells. CTLA4 plays a central role in maintaining immune tolerance by regulating T cell co-stimulation at the time of their initial response, thus counterbalancing the effect of T-cell receptor/CD3 activation and CD28 costimulation signals. CTLA-4 was the first immune-checkpoint molecule to be clinically targeted: ipilimumab became the first successfully developed anti-CTLA-4 drug and was approved in 2011 for the treatment of advanced or unresectable melanoma, displaying long-term survival [39]. The clinical activity of ipilimumab in combination with fotemustine was shown in melanoma patients with brain metastases [39]. Similarly, inhibitors of PD-1, another checkpoint target expressed on activated T-cells and mediating immunosuppression via the binding to its ligands (PD-L1 and PD-L2), nivolumab and pembrolizumab have been approved to treat patients with advanced or metastatic melanoma and patients with metastatic, refractory non-small cell lung cancer.

CheckMate 067 trial results indicate significantly longer OS results for nivolumab combined with ipilimumab vs nivolumab alone in patients with previously untreated advanced melanoma [40]. A phase III study (CheckMate 238) is currently ongoing for understanding the efficacy of adjuvant immunotherapy with nivolumab versus ipilimumab after complete resection of stage IIIb/c or stage IV melanoma in patients at high risk recurrence.

Immune checkpoint therapy is also being considered for the treatment of mesothelioma, a rare, aggressive form of cancer that develops in the lining of the lungs, abdomen, or heart [41, 42]. The monotherapy with tremelimumab, an anti-CTLA-4 inhibitor, especially in an intensified schedule, has shown clinical and immunological activity in patients with advanced malignant mesothelioma (52% of patients achieved disease control, with a median duration of 10.9 months) and a good safety profile [42, 43]. However, the same treatment failed to significantly prolong OS in patients with relapsed malignant mesothelioma, having a safety profile consistent with the known CTLA-4 inhibitors [44].

Other investigations are ongoing to assess whether combination regimens provide greater efficacy in mesothelioma [i.e. Phase II study of tremelimumab combined with the anti-PD-L1 drug durvalumab, having already proved the safety and manageability of the drug combination (NCT02588131)]. Moreover, a Phase III trial is evaluating the combination of nivolumab plus ipilimumab vs pemetrexed and cisplatin or carboplatin as first-line therapy (CheckMate 743).

Although immune-checkpoint inhibitors showed a favorable efficacy/toxicity profile with durable response in different cancer types, no predictive biomarkers have been yet validated to select patients potentially benefitting from therapy.

Inducible T cell co-stimulator (ICOS) is being evaluated as a pharmacodynamic biomarker associated with survival and/or clinical benefit in melanoma treated with ipililumab. The use of GSK3359609, an ICOS agonist antibody is under evaluation alone and in combination with other cancer immunotherapies such as pembrolizumab in selected advanced cancers (NCT02723955). Among eventual predictive biomarker candidates, the mismatch repair status of the tumor has been considered and investigated in tumors treated with PD-1 blockade [45]. It could predict clinical benefit of immune checkpoint blockade with pembrolizumab in progressive metastatic carcinoma.

Already approved by both the FDA and EMA for multiple types of tumors, i.e. advanced melanoma, non-small cell lung cancer, head and neck cancer, Merkel cell carcinoma, renal cell carcinoma and hematological malignancies, immune checkpoint inhibitors also appear to have significant antitumor activity in multiple other tumor types, however not all patients benefit from therapy. Efforts should focus on improving the efficacy of immunotherapy through the use of combination approaches for synergistic effects, and predictive biomarkers of response and resistance.

### Addressing cancer responsiveness to immunotherapy

Independently of cancer histology, the effectiveness of cancer immunotherapy is clearly limited to a subset of patients with inflamed tumors [46]. Based on the level of T-cell presence and activity, three primary immune phenotypes within the tumor microenvironment can be identified: immune desert (no cancer immunity), stromal excluded (active T-cells unable to reach tumor cells), and inflamed (active T-cells in tumor microenvironment not functioning properly). However, the number of hypotheses explaining the resistance to immunotherapies exceeds the above-recognized tumor immune landscapes. Thus, there is a need for a unifying theory to explain the response patterns with their resistance mechanisms. Dr. Marincola and his collaborators developed the “theory of everything”, which assigned resistance mechanisms to a specific immune landscape according to its transcriptional expression pattern. Thus, cancers can be defined according to their immune contexture with new taxonomies derived from global transcriptional patterns [47, 48]. For example, global transcription analyses of needle aspiration samples from melanoma metastases from patients undergoing immunotherapy showed that half of the genes predictive for clinical response were related to T-cell regulation, suggesting that immune responsiveness might be predetermined by a tumor microenvironment conducive to immune recognition [49]. Interestingly, the monitoring of the T-cell inflamed gene expression profiles has shown that responsiveness to checkpoint inhibitor therapy is observed almost exclusively in immune-active landscape containing IFN-γ—responsive genes related to antigen presentation, chemokine expression, cytotoxic activity, and adaptive immune resistance and correlating with clinical benefit [50]. The theory of immune contexture is characterized by immune signatures associated with the immunologic constant of rejection (ICR), which comprises a set of genes indicating an active immune engagement and at least a partial rejection of the cancer tissue [51]. However, the immune-active landscape is not sufficient alone to predict immune response. Indeed, other factors are influencing the immune response: the host’s genetics, the tumor’s genetics and the microenvironment [52, 53]. A bioinformatics approach has been used for optimal stratification of patients with breast cancers who may benefit from immune checkpoint inhibitors. Immune cell-specific gene signatures discriminate between immune benefit-enabled and immune benefit-disabled tumors [54]. Later, a clustering based on ICR genes segregates breast cancer patients into four different groups: ICR1, 2, 3 and 4. ICR4 corresponds to patients having the highest expression of the ICR gene signature and have a better prognosis compared to other ICR groups [55]. RNA-sequencing data from the TCGA consortium were used to define cancer immune phenotypes in breast cancer patients. In addition, somatic mutations and copy number alterations were mined to capture genetic features associated with such immune phenotypes. With this approach, it has been showed that MAPK modulation might enhance the therapeutic efficacy of immunomodulatory approaches in breast cancer. Interestingly, these main findings were validated in an independent meta-cohort of breast cancer samples [55]. Subsequently, by mapping gene signatures according to their expression in different immune landscapes of breast, lung, colon cancers, and melanoma, the theory of the “two option choice” was stipulated. This theory proposes that tumors evade recognition by the immune competent host by either adopting a “clean” oncogenic process, free of immunogenic stimuli (immune silent tumors) or, display a profile that tends towards immune recognition (immune active tumors) but resists rejection by recruiting compensatory immune suppressive processes. Thus, immunotherapy is likely to be most successful in targeting immune active tumors, while silent tumors should be approached with only therapeutic manipulations that affect the intrinsic cancer cell biology in order to be clinically successful.

### Molecular targeted therapies: JAK inhibitors in autoimmune and inflammatory diseases

Cytokines are pivotal in the maintenance of an appropriate immune response. Dysregulation of cytokine activity results in the loss of the immune system homeostasis, and the targeting of cytokine receptors has been an effective means of treating immune-related disorders. In the last few years, research efforts have been directed towards cytokines’ intracellular signaling pathways. Upon binding to their specific receptor, cytokines trigger a cascade of events involving activation of the tyrosine kinase of the Janus family, also known as JAKs, as the first and critical step.

Inhibition of JAK enzymatic activity has proved successful for some immune-mediated pathologies. So far, four JAKs inhibitors have been approved for clinical use. Ruxolitinib is a JAK2/JAK1 inhibitor currently utilized for the treatment of myeloproliferative disorders.

Tofactinib, a JAK3/JAK1 inhibitor, is approved for the treatment of rheumatoid arthritis, psoriatic arthritis and ulcerative colitis. It was recently shown that one of JAK3/JAK1 inhibitors, tofacitinib, is efficacious in ameliorating several aspects of the pathology associated with a murine model of Systemic Lupus Erythematosus and a Phase Ib trial in Lupus patients was recently concluded at National Institutes of Arthritis Musculoskeletal and Skin Diseases (NCT02535689).

Moreover, JAK inhibitors are being investigated for several other pathologies including alopecia, vitiligo, psoriasis, inflammatory bowel disease (IBD), atopic dermatitis as well as for genetically-inherited diseases characterized by overproduction of Interferons.

Interestingly, only mild toxicity was observed in patients treated with these drugs even if these drugs target several cytokines, including interferons, hormones and growth factors. Side effects included increased rates of infections, low hemoglobin levels, and augmented circulating lipoproteins, creatinine and transaminases. The blockade of a single JAK could potentially reduce toxicity for these drugs, especially in the setting of long-term administration. Therefore, more selective JAK inhibitors are currently being developed and are currently being tested. If the increased selectivity will affect their efficacy remains to be seen.

## Cell therapy and adoptive cell therapy

### Mesenchymal stromal cells (MSCs)

Mesenchymal stromal cells display immunoregulatory and regenerative properties [56]. The relative ease of harvesting MSCs, and their stable phenotype upon in vitro culture, render MSCs an attractive tool for cell-based therapy in alloimmunity, autoimmunity and inflammation. This is reflected by the increased numbers of published clinical trials—for example, in GvHD [57], Crohn’s disease (CD) [58] and multiple sclerosis [59]—and of ongoing clinical trials.

MSCs are non-hematopoietic cells expressing different surface molecules (including CD90, CD73, CD105, CD29, CD44, and CD166), but lacking the expression of endothelial or hematopoietic markers (CD31, CD45, CD43, CD14, CD11b), major histocompatibility complex (MHC) class II molecule, and costimulatory proteins (CD80, CD86, CD40) [60, 61].

MSCs display low engraftment rates after therapeutic infusion, and the engraftment is not directly proportional to response to treatment. These cells might carry out their functions through a “hit and run” mechanism, while limiting eventual therapy related long-term risks with their short persistence [62].

Upon in vitro culture, and -although to a lesser extent- in in vivo models, MSCs trigger the instant blood mediated inflammatory reaction (IBMIR). The activation of coagulation and complement cascades may activate immune responses. Induction of IBMIR is dose-dependent and increases after prolonged ex vivo expansion: cell doses of low-passage clinical-grade MSCs currently administered in clinical trials elicit only minor systemic effects [63].

The immunomodulatory properties of MSCs, through the down-modulation of inflammatory responses, the increase of T-reg cell frequencies and the enhancement of tissue repair and homeostasis, represent the rationale for their application in several clinical settings [57, 64, 65], including intestinal inflammation in both acute GvHD and IBD.

Despite the availability of a wide range of therapeutic strategies, the management of severe and refractory cases of IBD represents a big clinical challenge. In this arena, MSCs stand out as a new therapeutic option. The demonstration that biological characteristics of the MSCs derived from CD patients are similar to those derived from healthy donors [66] has represented the basis for an autologous approach of anti-inflammatory/reparative cell therapy in patients with refractory CD.

In a Phase I/II study, the intrafistular injection of autologous bone marrow-derived MSCs (BM-MSCs) from patients with refractory fistulizing CD revealed to be safe and effective, obtaining a significant reduction of both Crohn’s Disease Activity Index (CDAI) and Perianal Disease Activity Index (PDAI). Seventy percent of the patients experienced complete and sustained healing of fistula tracks, and 30% a partial response [67].

In a Phase I study it has been observed that the intravenous injection of autologous MSCs for refractory luminal CD has an optimal safety profile and efficacy in 4 out of 10 treated patients [58]. Recently, a multicenter Phase III randomized study compared the efficacy of third-party adipose tissue-derived MSCs (AT-MSCs) (industrial preparation) with a placebo. MSCs were injected intra-lesion in refractory fistulizing CD patients. The primary endpoint combining both clinical assessment of fistula closure and MRI found 50% combined remissions 24 weeks after treatment in the MSC group vs 34% in the placebo group [68]. Autologous/3rd party BM-/AT-MSCs therefore provide an effective therapy for patients with refractory fistulizing CD who do not respond to conventional and/or biological treatments.

### Adoptive cell therapy

Antigen specific T-cell therapies contributed to ameliorate the haplo-transplant outcome in the last decade, which saw a drastic reduction in transplant-related mortality: 6% after 2005 vs 33% before 2005 (Pavia cohort, unpublished data). Adoptive T-cell therapy is useful in reconstituting specific immunity and in the treatment of Epstein–Barr virus (EBV)-related post-transplant lymphoproliferative disorder (PTLD) [69, 70], HHV6-related transplant-rejection, severe human Cytomegalovirus-related colitis in patients with primary immunodeficiency [71], polyomavirus BK infections [72], and progressive multifocal leukoencephalopathy associated with polyomavirus JC [73]. Remaining problems related to the implementation of this approach are: (i) producing viral-specific T-cells targeting simultaneously multiple viruses, (ii) reducing production time, and (iii) producing biological agent with efficacy in recipients of seronegative donors [74].

Several attempts have already been done in those directions, such as monoculture-derived multi-specific cytotoxic T-lymphocytes (CTL) against CMV and EBV, several serotypes of adenovirus that were proven to be safe and efficient means to restore virus-specific immunity in the immunocompromised host [75, 76], and third-party EBV-specific CTLs for PTLD, which showed 42% complete remission (CR) at 6 months and 70% overall survival at 2 years, although long-term results were less satisfactory [77–79].

Moreover, adoptive cell therapy has also been implemented for cancer treatment. Cell therapy with EBV-targeted autologous CTLs was shown to be safe, capable of inducing specific immunologic responses, and associated with objective responses and control of disease progression in patients with stage IV nasopharyngeal carcinoma resistant to conventional treatments [80]. The results are encouraging, although further improvements to the clinical protocols are clearly necessary to increase anti-tumor activity, and promising implementations are underway, including harnessing the therapeutic potential of CTLs specific for subdominant EBV latent cycle epitopes, and delineating strategies aimed at targeting immune evasion mechanisms exerted by tumor cells [81]. Another recently developed application is in the context of Philadelphia chromosome-positive acute lymphoblastic leukemia (Ph^+^ ALL): 3 treatment-refractory patients were treated with autologous or allogeneic p190BCR-ABL-specific CTLs, achieving molecular or hematologic CR upon the emergence of p190BCR-ABL-specific T-cells in the bone marrow [82].

## Diabetes

Diabetes is a metabolic disorder that is characterized by chronic hyperglycemia due to progressive loss of pancreatic β-cells, which, over time, can lead to several well-recognized and debilitating complications. The Qatar National Diabetes strategy 2016–2022 stated that diabetes is a major health challenge with a prevalence that is over double that of the world population, at 17%. To date, there is no permanent treatment available for diabetes. Cell therapy might prove to be the best approach to treat type 1 diabetes (T1D), monogenic diabetes, and severe cases of type 2 diabetes (T2D).

### Cell-based immunomodulation: MSCs

Mesenchymal stromal cells are considered one of the most interesting candidates for the treatment of T1D based on their anti-inflammatory and immunomodulatory capacities, associated with poor immunogenicity that enables them to escape the host immunesurveillance. Several pre-clinical studies, in streptozocin-induced diabetic mouse models, showed that MSCs protect from diabetes or revert the diabetic phenotype [83–86]. A Phase I clinical trial was conducted in Sweden, where adult patients recently diagnosed with T1D received autologous BM-MSCs. During the first year after diagnosis, a preserved or even increased C-peptide response to a mixed-meal tolerance test in treated patients was observed, while no adverse events were registered [87]. Apparently, MSCs derived from T1D patients, despite some clear transcriptional differences, do not demonstrate a significant difference from healthy controls in immunosuppressive activity, migratory capacity, or hemocompatibility [88].

### Beta-cell replacement

Insulin replacement for T1D is burdened by relatively low clinical efficacy, low compliance to glycemic control (roughly about 1/4 of patient with type 1 reaches therapeutic targets) [89], high and increasing cost (about 3% increase/year), and high impact on quality of life [90–92] thus requiring alternative approaches. Beta-cell replacement can be obtained from different sources, such as transplantation of whole pancreas or pancreatic islets, pancreatic duct stem cells, α-cells, human embryonic stem cells (hESCs) and induced pluripotent stem cells (hiPSCs), and β-cell expansion both in vivo and ex vivo [93].

### Islet transplantation

Transplantation of islets of Langerhans consists of implantation of different degrees of purified endocrine pancreatic tissue in the recipient’s hepatic portal system. The transplantation allows the restoration of the functional β-cells in diabetic patients [94], halting the late complications of diabetes, namely vasculopathy, retinopathy, nephropathy and neuropathy, in the face of a prolonged immunosuppressant therapy. From 1989 to 2017, 211 patients were transplanted at San Raffaele Scientific Institute, Italy (which ranks 2nd in the world for this procedure), granting patients long term insulin independence [94]. However, organ shortage, uncertain risk/benefit balance (i.e. absence of clear indications, absence of functional outcome definition, anti-rejection drugs, surgical risk, and long-term success rates) and financial barriers represent the major limitation for the extensive application of the procedure.

### Pluripotent stem cells

Stem cell therapy is becoming a concrete opportunity to treat various diseases. In particular, for a disease like T1D, caused by the loss of a single specific cell type that does not need to be transplanted back in its originating site to perform its function, a stem cell-based cell replacement therapy seems to be the ideal cure. ViaCyte (formerly Novocell) has optimized the protocol for making pancreatic progenitors from human pluripotent stem cells (hPSCs) [95, 96]. A clinical trial for diabetic patients treated with human embryonic cells (hESC)-derived pancreatic progenitors macroencapsulated in ENCAPTRA is currently ongoing at San Raffaele Scientific Institute, Italy, in collaboration with Viacyte (US), Brussels University (Belgium), Leiden University (Netherlands) and Nestlè Institute of Health Sciences (Switzerland) (http://viacyte.com/clinical/clinical-trials/). Before transplanting those progenitors, the co-expression of two transcription factors PDX1 and NKX6.1 must be confirmed to assure their differentiation inside the body within few weeks into islet cells [97]. Recently, Abdelalim’s group was able to establish a highly efficient protocol to generate around 90% of hPSC-derived pancreatic progenitors expressing the key transcription factors (PDX1^+^/NKX6.1^+^) required for β-cell maturation [98, 99], indicating a dramatic improvement in the differentiation process. Interestingly, a novel pancreatic progenitor population has been recently generated from hPSCs, expressing NKX6.1 but not PDX1 (PDX1^−^/NKX6.1^+^). The characterization of this novel population showed pancreatic endocrine precursor features, defining it as a potential new source for pancreatic β-cells [98, 99]. Furthermore, hPSCs, including hESCs and human induced PSC (hiPSCs) can provide a renewable source of functional pancreatic β-cells to study and treat diabetes, because hPSC-derived β-cells can be closer in nature to the in vivo β-cells than those differentiated from other types of stem cells. hiPSC technology allows to directly generate hPSCs from diabetic patients, providing cells genetically identical to patients to be used for in vitro disease modeling and eventually cell-based therapies [97]. In Qatar, Abdelalim’s team has generated several hiPSC lines from insulin resistant and diabetic patients, with in vitro differentiation capacity into different cell types including β-cells and insulin-target cells that can be used to understand the pathophysiology of the diabetes.

### Continuous subcutaneous insulin infusion using computerized program

A pilot study is ongoing in Sidra Medicine, Qatar, aiming to evaluate the effectiveness of a unified computerized program for the initiation of continuous subcutaneous insulin infusion (CSII) in T1D patients in Qatar. CSII settings were performed using a specific computerized program (built in-house), calculating basal rates, bolus wizard and sensor settings, once entered with age, HbA1c, basal/bolus insulin dose, wake-up and school time for each patient.

A significant increase of total insulin dose by 27% and significant decrease of HbA1c by 1.5% was detected at the end of the study.

Although conducted on a small cohort (34 patients) and a short follow-up period (6 months), the study showed that unified computerized program for CSII initiation may improve glucose control in T1D patients.

## Advanced therapeutic techniques

### Thymus transplantation

Thymus transplantation is a promising strategy for the treatment of athymic complete DiGeorge syndrome (cDGS), characterized by profound T-cell deficiency and associated with a hemizygous microdeletion at chromosome 22q.11, CHARGE (Coloboma, heart defects, atresia choanae, retardation of growth and development, genital abnormalities, ear abnormalities/deafness) syndrome, mutations in TBX1, deletions at chromosome 10p13-14, or fetal toxin exposure from glucose, ethanol, or retinoic acid.

The survival of patients with this anomaly is usually low, with most patients dying before the age of 2 years. Bone marrow or peripheral T-cell transplant in Di George patients seems to slightly improve the survival rate [100], whilst thymus transplantation significantly increases the survival rate, with one study reporting the survival of over 60% (n = 60) of patients surviving over 15 years post-transplant [101]. Eighteen patients with cDGS underwent transplantation with allogeneic cultured thymus: 2 patients died early of pre-existing viral infections, and 1 late death occurred from autoimmune thrombocytopenia. Evidence of thymopoiesis developed at 5–6 months after transplantation in 14 patients, who were able to clear pre-existing infections and those acquired later. Autoimmune complications were seen in 8 of 18 patients, including thyroiditis, hemolysis, thrombocytopenia, and neutropenia.

In the future, a combined approach of newborn screening, allowing earlier recognition of the disease and less infections, and thymus cryopreservation for a timely readiness and better HLA matches, could eventually improve the survival rate.

### Intrauterine surgery

Intrauterine surgery is an intervention performed on a fetus while still in utero, with the goal to correct an alteration in the normal fetal development. This cutting-edge procedure considers the fetus not merely a tissue, but a patient, inside of another patient: the mother.

More than a simple surgery, it is rather considered “an enterprise”, including a multidisciplinary involvement of teams collaborating in the same operating theater: surgery, perinatology, neonatology, anesthesiology, cardiology, and interventional radiology. Both life-threatening fetal diseases (such as cystic adenomatoid malformation, sacrococcygeal teratoma, twin-twin transfusion syndrome, valvular obstruction, urinary obstruction) and non-life-threatening complications, (e.g. Fetal Myelomeningocele or Open Spina Bifida) can be addressed with this technique.

Myelomeningocele is a degeneration of the neural tube. In early gestation, the neural tube is open but has a normal cytoarchitecture, while neuronal deterioration occurs later in utero, coming from the intrauterine environment. Ultrasound reports show good leg movements of the fetus at 16 weeks, but deterioration by birth. Strikingly, a randomized trial comparing prenatal vs postnatal Myelomeningocele surgery was stopped for efficacy in the prenatal arm [102]. Another randomized trial showed how intrauterine treatment of patients with high risk severe isolated congenital diaphragmatic hernia grants a significant survival benefit for the newborns with good maternal outcomes [103].

## Abbreviations

AAV: adenoviral vector
AT-MSCs: adipose tissue-derived MSCs
ATH: Advanced Therapies in Healthcare
BM-MSCs: bone marrow-derived MSCs
BMT: bone marrow transplant
CDAI: Crohn’s Disease Activity Index
CSPG4: chondroitin sulfate proteoglycan 4
CTL: cytotoxic T lymphocyte
CTLA-4: cytotoxic T-lymphocyte antigen 4
EBV: Epstein–Barr virus
EGFR: epidermal growth factor receptor
FDA: Food and Drug Administration
GBM: glioblastoma multiforme
GVL: graft versus leukemia
hESCs: human embryonic stem cells
hiPSCs: human induced pluripotent stem cells
HSCT: hematopoietic stem cell transplant
IBD: inflammatory bowel disease
IBMIR: instant blood mediated inflammatory reaction
ICOS: inducible T cell co-stimulator
MHC: major histocompatibility complex
MSCs: mesenchymal stromal cells
NK: natural killer
OS: overall survival
PDAI: Perianal Disease Activity Index
PTLD: post-transplant lymphoproliferative disorder
T1D: type 1 diabetes
T2D: type 2 diabetes
TME: tumor microenvironment
